# Characterization of Fusarium Species and Soil Herbicide Effects on Fusarium graminearum in Maize Fields of Eskişehir, Türkiye

**DOI:** 10.3390/plants15081254

**Published:** 2026-04-19

**Authors:** Semir Turuşkan, Filiz Ünal

**Affiliations:** Department of Plant Protection, Faculty of Agriculture, Eskişehir Osmangazi University, Eskişehir 26040, Türkiye; semir_turuskan@hotmail.com

**Keywords:** *Fusarium graminearum*, soil herbicides, maize, pathogenicity, *in vitro* and *in vivo* assays, plant growth, disease management

## Abstract

To determine *Fusarium* species and their pathogenicity in maize-production areas of the Tepebaşı, Odunpazarı, Alpu, and Seyitgazi districts of Eskişehir province, Türkiye, 180 samples were collected from 45 fields during survey studies conducted in 2023–2024. A total of 110 *Fusarium* isolates were obtained from the collected plant samples. The isolates were identified as *F. verticillioides*, *F. culmorum*, *F. proliferatum*, *F. graminearum*, *F. sambucinum*, *F. acuminatum*, *F. chlamydosporum*, and *F. equiseti*. The most common species was *F. verticillioides*, while the most virulent species was *F. graminearum*, with a disease severity of 96.67%. The effects of different doses of soil-applied herbicides containing the active ingredients Isoxaflutole + Thiencarbazone-methyl + Cyprosulfamide, Dimethenamid-P + Saflufenacil, and S-Metolachlor + Terbuthylazine on *F. graminearum* were evaluated under both *in vitro* and *in vivo* conditions. Under *in vitro* conditions, the highest inhibition rate (57.23%) was observed in the double-dose application of the herbicide containing S-Metolachlor + Terbuthylazine. This was followed by the upper and recommended doses of the same herbicide with inhibition rates of 47.16% and 39.46%, respectively. For the other herbicides, inhibition rates increased with increasing herbicide dose. In field trials, the highest suppression of the pathogen was also observed with the herbicide containing S-Metolachlor + Terbuthylazine. While the recommended dose showed a 38.6% effect against the pathogen, the upper dose resulted in a 45.31% effect. This study suggests that herbicide applications may be associated with improved plant growth, likely due to reduced pathogen pressure and decreased weed competition. The findings highlight the complex interactions between soil-applied herbicides, soil-borne pathogens, and host plants, and provide insights into the development of integrated disease management strategies in maize-production systems.

## 1. Introduction

Maize (*Zea mays* L.) is a cross-pollinated, monoecious, short-day plant. The male and female flowers are located at different positions on the same plant. Due to this characteristic, maize is classified as a monoecious species [1]. Maize is currently one of the six major cereal crops that sustain the world’s population. In addition, owing to its versatile uses, high adaptability, and productivity, maize is the second most important cereal crop worldwide after wheat [2,3]. Maize is native to the American continent, from which it spread throughout the world [4]. The introduction of maize into Türkiye occurred via North Africa. The designation of this crop as “mısır” in Turkish is considered an indication that it entered the region through Egypt and Syria [5,6]. Globally, including Türkiye, maize varieties are commonly classified into seven kernel-based types: dent maize, flint maize, popcorn, sweet corn, pod corn, flour (soft) maize, and waxy maize. Among these, dent maize and flint maize are the most widely cultivated types. In Türkiye, dent maize is predominantly used for animal feed, either as grain or for silage production [7]. According to official agricultural statistics, global maize production in 2024 reached approximately 1.2 billion tonnes [8]. In Türkiye, maize production declined to about 8.1 million tonnes in 2024, making it the third most produced cereal crop after wheat and barley [9]. Maize is used not only for human consumption but also extensively as animal feed and as an industrial raw material; the whole grain and its processed fractions such as starch, oil, and proteins are important products for food and industrial applications [10,11,12]. In addition, a significant portion of maize biomass, including stalks and leaves, is also used as forage or silage for livestock [13].

Maize diseases, particularly fungal diseases affecting the roots, crown, stalks, and ears, are among the most important factors limiting yield [14]. *Fusarium* spp. are among the most destructive pathogens affecting maize production worldwide. Stalk and ear rot diseases, which cause significant yield losses in maize, are primarily caused by *Fusarium* spp. [15,16]. Under favorable environmental conditions, these fungi can cause severe yield losses, which may reach up to 50–70%, depending on climatic conditions during the growing season, cultivar susceptibility, plant developmental stage, and the occurrence of mechanical injuries. In addition to direct yield reductions, the ability of *Fusarium* spp. to produce a wide range of mycotoxins has further increased the significance of these pathogens in maize-based production systems. Among these mycotoxins, fumonisins are the most frequently reported and economically important in maize; however, other *Fusarium*-derived toxins such as deoxynivalenol (DON), zearalenone (ZEA), and emerging mycotoxins including beauvericin and moniliformin may also be present. These compounds pose serious risks to both human and animal health due to their toxic, carcinogenic, or endocrine-disrupting effects. Previous studies have demonstrated that late-season rainfall and high humidity during maize cultivation promote *Fusarium* infection and enhance mycotoxin accumulation, both in the field and during post-harvest drying and storage processes [17,18,19]. The most important *Fusarium* spp. causing stalk, ear, and kernel rots in maize are *F. verticillioides* and *F. graminearum*. *Fusarium* spp. are both seed-borne and soil-borne pathogens. These diseases, particularly those affecting ears and kernels, directly reduce yield in maize. *Fusarium* spp. invade the plant’s vascular tissues, leading to systemic infection. In maize, infection typically occurs through roots, wounds, or silks, after which the pathogen colonizes cortical tissues and progresses into the vascular system, allowing it to spread systemically within the plant. This process may result in stalk rot, ear rot, and kernel infection, particularly under favorable environmental conditions [15,17]. Consequently, effective management of the diseases caused by these pathogens is highly challenging. It is crucial to initiate control measures before disease development or immediately after the first visible symptoms appear, as delayed interventions often result in failure. Therefore, effective control of mycotoxin-producing pathogens is also essential to ensure food safety [20].

The increasing use of pesticides in agricultural production has raised concerns about unintended consequences, particularly the repeated application of different pesticide formulations on the same agricultural land within a single growing season [21]. In maize-production systems, pre-emergence and post-emergence herbicides are widely used for weed control. Nevertheless, information regarding the effects of pre-planting or pre-emergence herbicides on soil- and seed-borne pathogenic fungi remains limited. Because *Fusarium* spp. are predominantly soil-borne pathogens that infect maize through root and lower stem tissues, soil-applied herbicides commonly used in maize-production systems were selected in this study. As these herbicides are applied directly to the soil before or shortly after planting, they may influence pathogen survival in the rhizosphere and affect host susceptibility during early plant development [15,16]. Based on this rationale, we hypothesized that soil-applied herbicides may influence the development of *Fusarium* infections and maize plant growth by altering rhizosphere conditions and plant physiological responses. Therefore, the aim of this study was to determine the distribution and pathogenicity of *Fusarium* spp. in maize-production areas of Eskişehir and to evaluate the effects of commonly used soil-applied herbicides on the development of *F. graminearum* under both *in vitro* and *in vivo* conditions.

## 2. Results

### 2.1. Survey and Sampling of Maize Fields

Survey studies were conducted in maize-production areas of Eskişehir province. Samples were collected from fields according to their size. A total of 180 samples were optained from 45 fields, including 45 samples from 11 fields in Seyitgazi, 65 samples from 16 fields in Alpu, 50 samples from 13 fields in Odunpazarı, and 20 samples from 5 fields in Tepebaşı.

### 2.2. Isolation and Identification of Fusarium spp.

In the survey conducted in maize production fields in Eskişehir province, samples collected from the neighborhoods of Merkez, Fevzipaşa, Sevinç, Ağapınar, Emirler, Karahöyük, Kızılcaören, 71 Evler, Koyunlar, Aksaklı, Kalkanlı, Büyükdere, Yenikent, Türkmentokat, Yazıdere, and Yunus, which are located in the districts of Tepebaşı, Odunpazarı, Seyitgazi, Alpu, and Çifteler, yielded a total of 110 *Fusarium* isolates. Morphological identification based on single-spore isolates revealed that the species obtained from maize fields included *F. verticillioides*, *F. culmorum*, *F. proliferatum*, *F. graminearum*, *F. sambucinum*, *F. acuminatum*, *F. chlamydosporum*, and *F. equiseti*. The most frequently isolated species was *F. verticillioides* with 33 isolates, followed by *F. culmorum* with 26 isolates, *F. graminearum* with 24 isolates, and *F. proliferatum* with 13 isolates. The least frequently isolated species were *F. sambucinum* and *F. chlamydosporum*, each with a single isolate.

*F. verticillioides*, *F. culmorum*, *F. proliferatum*, *F. graminearum*, and *F. acuminatum* were isolated from all districts, whereas *F. sambucinum* was found only in Alpu, *F. chlamydosporum* only in Odunpazarı, and *F. equiseti* in both Odunpazarı and Seyitgazi. Overall, the most widespread species was determined to be *F. verticillioides*. The isolates of *F. verticillioides*, *F. culmorum*, *F. proliferatum*, and *F. graminearum*, which were the most frequently obtained species in the classical identification studies, were also subjected to molecular identification to confirm the species identification (Table 1). Among the obtained isolates, 41 representative isolates were selected for pathogenicity tests based on species distribution and isolate characteristics.

### 2.3. Pathogenicity Tests

The pathogenicity of 41 selected *Fusarium* isolates was tested under greenhouse conditions using the susceptible maize cultivar PL538, and disease severity values were calculated. All tested *Fusarium* isolates were found to be pathogenic. In greenhouse pathogenicity tests, the inoculated maize plants developed typical symptoms associated with *Fusarium*-induced root and crown rot, including hypocotyl and crown infections, reduced root length, and stunted plant growth. The most virulent isolate was identified as *F. graminearum* with the code M14, showing a disease severity of 96.67%. This was followed by the *F. culmorum* isolate M53 X, with a disease severity of 93.33%. The disease severity values of *F. verticillioides* isolates ranged from 60% to 86.67%, while the highest disease severity for *F. proliferatum* isolates was 84%. The least virulent isolates were M31 Z1 (*F. culmorum*) and M33 X (*F. verticillioides*), each showing a disease severity of 60% (Table 1). The regions with the highest disease severity were identified as the central Alpu area and the 71 Evler neighborhoods in Tepebaşı. Based on these findings, field experiments were conducted to further evaluate the effects of soil-applied herbicides on the most virulent isolate, M14 (*F. graminearum*). Values represent the mean of three replicates (mean ± standard error, *n* = 3). Different letters indicate statistically significant differences among the values according to Tukey’s test at *p* < 0.001.

### 2.4. Determination of the Effects of Herbicides

#### 2.4.1. Effects of Herbicides on Mycelial Growth of the Pathogen Under *In Vitro* Conditions

The effects of herbicides on the most virulent isolate, M14 (*F. graminearum*), were evaluated under *in vitro* conditions on Potato Dextrose Agar (PDA) medium. Soil-applied herbicides with the active ingredients Isoxaflutole + Thiencarbazone-methyl + Cyprosulfamide (herbicide 1), Dimethenamid-P + Saflufenacil (herbicide 2), and S-Metolachlor + Terbuthylazine (herbicide 3) were applied at the recommended dose, lower dose, higher dose, and double dose. The data obtained from Petri dish experiments evaluating the effects of herbicide active ingredients and their doses under *in vitro* conditions on both measurement days (2nd and 3rd day) were subjected to analysis of variance according to a factorial experimental design. Results found to be significant at *p* ≤ 0.05 were further compared using Duncan’s Multiple Range Test and classified according to their significance levels.

The results indicated that all tested herbicides generally had a significant effect on the mycelial growth of the pathogen compared to the untreated control. Dimethenamid-P + Saflufenacil and S-Metolachlor + Terbuthylazine were found to significantly inhibit mycelial growth at even the lower doses on both measurement days. In contrast, the herbicide containing Isoxaflutole + Thiencarbazone-methyl + Cyprosulfamide showed a significant effect only from the higher dose onward on both measurement days. Statistically, all doses of S-Metolachlor + Terbuthylazine were more effective than all doses of the other two herbicides, indicating that this herbicide was the most effective active ingredient in suppressing fungal development. Overall, these results demonstrate that S-Metolachlor + Terbuthylazine exhibited the strongest inhibitory effect on the mycelial growth of *F. graminearum* under *in vitro* conditions.

Considering the inhibition rates of the pathogen at different herbicide doses, it was observed that increasing the dose generally led to higher inhibition for all herbicides. For the herbicide containing Isoxaflutole + Thiencarbazone-methyl + Cyprosulfamide, the highest inhibition in the first measurement was 17.87% at the double dose, while the effect at the recommended dose was calculated as 10.9%. In the second measurement, the inhibition rates for this herbicide were 16.67% and 8.54% at the respective doses, indicating a decrease in inhibition over time. For Dimethenamid-P + Saflufenacil, the highest effect in the first measurement was 30.87% at the double dose, with the recommended dose showing 21.94% inhibition. In the second measurement, the inhibition rates decreased to 22.54% and 17.42% for the respective doses. Overall, in the Petri dish assays, the effects of Isoxaflutole + Thiencarbazone-methyl + Cyprosulfamide and Dimethenamid-P + Saflufenacil on the pathogen were found to be relatively low. For the herbicide containing S-Metolachlor + Terbuthylazine, the highest effect in the first measurement was observed at the double dose, with an inhibition rate of 57.66%, while the effect at the recommended dose was calculated as 38.72%. In the second measurement, the inhibition rate at the double dose remained almost the same (56.78%), whereas the inhibition at the recommended dose increased slightly to 40.18%. The average inhibition rate of this herbicide against the pathogen was determined to be 57.22% (Table 2). Overall, the results indicate a dose-dependent inhibitory effect of the tested herbicides on *F. graminearum*, with S-Metolachlor + Terbuthylazine showing the highest inhibitory effect on fungal growth under *in vitro* conditions.

#### 2.4.2. Effects of Herbicides on Disease Severity and Plant Development Under *In Vivo* Conditions

The effects of different doses of herbicides containing Isoxaflutole + Thiencarbazone-methyl + Cyprosulfamide, Dimethenamid-P + Saflufenacil, and S-Metolachlor + Terbuthylazine on disease severity were investigated under field conditions. Disease severity assessments were based on root and crown rot symptoms observed at the 8–10 leaf stage, including root discoloration, reduced root length, and decreased plant growth, rather than wilt symptoms. In addition, the effects of these soil-applied herbicides on maize plant development parameters, including plant height, leaf number, leaf width, leaf length, root collar diameter, root length, and root weight, were evaluated.

Reductions in disease severity were observed in plants treated with different doses of the three herbicides under field conditions. In the untreated control plots, the disease severity caused by the pathogen was calculated as 88.27%. Among the herbicide-treated plots, the lowest disease severity (48.27%) was recorded with the highest dose of S-Metolachlor + Terbuthylazine. Furthermore, at the recommended dose of this herbicide, the disease severity (54.67%) was lower than that observed at all dose applications of the other two herbicides (Table 3).

An increase in efficacy values was observed with increasing doses for all herbicides. The highest efficacy was recorded for the herbicide containing S-Metolachlor + Terbuthylazine. For this herbicide, the maximum effect (45.31%) was observed at the highest dose, while the recommended dose exhibited an efficacy of 38.06%, and the lowest dose resulted in an efficacy of 25.37%. For Dimethenamid-P + Saflufenacil at the recommended dose, the effect was 27.49%, whereas for Isoxaflutole + Thiencarbazone-methyl + Cyprosulfamide at the recommended dose, the efficacy against the disease was 22.96% (Table 3).

Examination of disease symptoms in plants treated with the herbicide containing Isoxaflutole + Thiencarbazone-methyl + Cyprosulfamide revealed that at the lower dose, pathogen-inoculated plants exhibited not only reduced plant height but also stem thinning, decreased root density, and reduced root length. In the positive control plots, which received only the pathogen, disease severity was high, accompanied by brown necrosis in the roots, extreme reductions in plant height, pronounced stem thinning, markedly decreased root density, and substantially reduced root length. These symptoms are consistent with *Fusarium*-induced root and crown rot of maize. Under field conditions, infected maize plants exhibited characteristic root and crown rot symptoms, mainly consisting of root decay and crown tissue damage. In contrast, plants treated with the recommended and high doses of the herbicide in combination with the pathogen, that is, recommended dose + pathogen and high dose + pathogen treatments showed improved growth and lower disease progression compared to the positive control plants. This improvement may be associated with the inhibitory effect of the herbicide on pathogen development.

Normal plant development was observed in the recommended dose without pathogen and high dose without pathogen plots, whereas plant growth in the low dose without pathogen plots was lower compared to the negative control. This reduced growth was attributed to insufficient weed control due to the suboptimal herbicide dose. In the recommended dose with pathogen and high dose with pathogen treatments, reductions in plant development, plant height, and root growth were observed compared to the negative control (no treatment), due to pathogen pressure. However, these plants exhibited better growth than those in the positive control (pathogen only) plots, likely due to the herbicide’s inhibitory effect on the pathogen. In the positive control, disease severity was high, accompanied by brown necrosis in the roots, extreme reductions in plant height, pronounced stem thinning, markedly decreased root density, and substantially shortened root length. In contrast, plants in the negative control (no herbicide, no pathogen) plots showed normal growth.

Examination of disease symptoms in plants treated with the herbicide containing Dimethenamid-P + Saflufenacil revealed that, despite pathogen inoculation, plants in all pathogen-inoculated plots exhibited better growth, plant height, and root development compared to the positive control plots. This improvement may be associated with the inhibitory effect of the herbicide on pathogen development. Plants in the negative control (NC: no herbicide, no pathogen) plots showed normal growth. Additionally, plant heights in the high-dose plots were observed to be lower than those in the recommended-dose plots, suggesting that high herbicide doses may slightly reduce plant growth. In the low-dose applications of S-Metolachlor + Terbuthylazine, a comparison between pathogen-inoculated and non-inoculated plots suggests that the slight reductions in plant height observed in the low-dose pathogen-inoculated plots were caused by the pathogen. The fact that plants in the low-dose pathogen-inoculated plots were in better condition than those in the positive control plots is also attributed to the effect of the herbicide. Severe disease symptoms and pronounced reductions in plant height in the positive control plots were solely due to the pathogen, and the poorer performance of positive control plants compared to herbicide + pathogen-treated plants supports this conclusion. The improved growth of plants in herbicide + pathogen plots, even at the low dose, can be explained by the herbicide’s inhibitory effect on pathogen development. This improvement is likely associated with reduced pathogen pressure and decreased weed competition resulting from herbicide application. However, due to the presence of the pathogen, plant development was generally reduced; consequently, plants in the herbicide + pathogen plots (low-dose pathogen-inoculated, recommendedrecommended-dose pathogen-inoculated, high-dose pathogen-inoculated) were shorter and exhibited weaker growth compared to those in pathogen-free plots.

In the recommended dose applications with and without pathogen, plant heights in the recommended-dose pathogen-inoculated plots were slightly lower, overall growth was weaker, and root density was reduced compared to the control. This reduction is likely due to the effect of the pathogen. However, plants in the recommended-dose pathogen-inoculated plots showed better growth and lower disease severity than those in the positive control plots, which is attributed to the herbicide’s inhibitory effect on the pathogen. In the high-dose applications, plants in the pathogen-free plots exhibited better growth than those in the high-dose pathogen-inoculated plots, likely due to the absence of the pathogen. Nevertheless, despite the presence of the pathogen, high-dose pathogen-inoculated plants performed better than positive control plants, indicating that the herbicide application may have contributed to reduced disease severity, thereby improving plant performance. Additionally, plant heights in the high-dose plots were observed to be shorter than in the recommended-dose plots, suggesting that high herbicide doses may slightly reduce plant growth.

The study results indicated that, across all herbicide dose applications, pathogen-inoculated plants (all doses + pathogen) exhibited better growth and reduced disease symptoms compared to positive control plants (pathogen only). This improvement may be associated with the inhibitory effect of the herbicide on pathogen development. Additionally, although weed populations were denser in the negative control (no herbicide, no pathogen) plots compared to all herbicide-treated plots, maize plants in these plots were observed to be healthier than plants in all low-dose, pathogen-free plots. This finding suggests that not only high herbicide doses but also low-dose applications can negatively affect plant growth.

### 2.5. Effects of Herbicides on Plant Development Under In Vivo Conditions

Field trials were conducted using the M14 (*F. graminearum*) isolate, which was the most virulent and commonly detected isolate as a result of pathogenicity and isolation studies.

#### 2.5.1. Plant Height

Based on the statistical evaluation of all sources of variation, significant differences were observed among disease status, herbicide type, and dose levels. Analysis of the effects of different herbicide doses on plant height indicated that, across all herbicide applications, plants in pathogen-inoculated plots exhibited shorter heights compared to those in pathogen-free plots.

When the statistical results were evaluated according to herbicide type, significant differences were observed in the effects of different active ingredients on plant height (Table 4). Considering pathogen–herbicide applications, the highest plant height (94.9 cm) was observed in the low-dose application of S-Metolachlor + Terbuthylazine (Herbicide 3). Since the average plant height in the control plot for this herbicide was 85.4 cm, the low-dose application resulted in an 11.2% increase in plant height. Following this, the low-dose application of Isoxaflutole + Thiencarbazone-methyl + Cyprosulfamide (Herbicide 1) resulted in an average plant height of 89.0 cm, which ranked second. Compared to the control, this represents a 4.21% increase in plant height. The recommended-dose application of the same herbicide (89.1 cm) was statistically in the same group as the low-dose treatment. The shortest average plant height (76.5 cm) was recorded in the recommended-dose application of Dimethenamid-P + Saflufenacil. Although plant heights for all doses of this herbicide were higher than their respective controls, they were shorter compared to the other two herbicide treatments.

**Figure 1:**
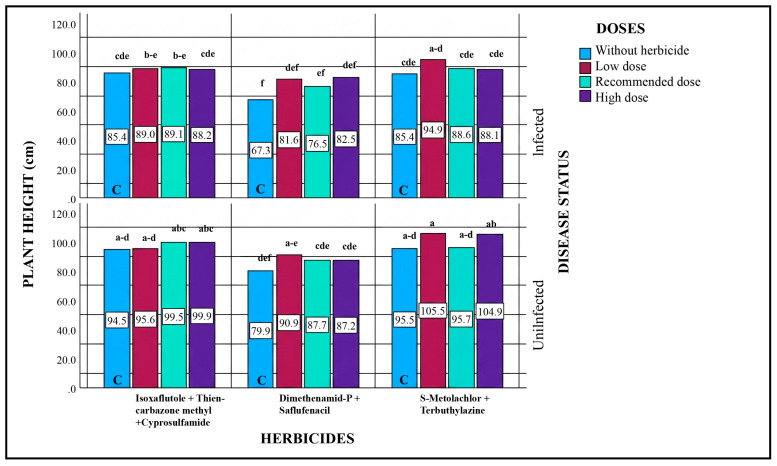
Effects of Different Herbicide Doses on Plant Height in Pathogen-Inoculated and Non-Inoculated Plots. Different lowercase letters indicate statistically significant differences according to Tukey’s test (*p* < 0.001). C indicates plots without herbicide application (with or without pathogen inoculation).

Considering the potential phytotoxic effects of herbicides, it is generally expected that increasing doses would suppress plant height, whereas plants in herbicide-free plots would be taller due to the absence of such stress. However, the results of this study showed the opposite trend: across all herbicides and in both pathogen-inoculated and non-inoculated plots, plant heights in herbicide-treated plots were higher than those in the untreated control plots (Figure 1). These findings suggest that, overall, all three herbicides, at all dose levels, reduced weed populations, which indirectly contributed to increased plant height.

#### 2.5.2. Leaf Width

**Figure 2:**
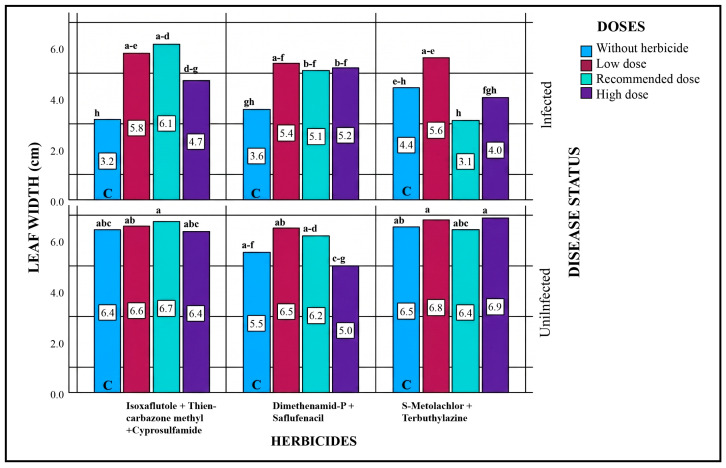
Effects of Different Herbicide Doses on Leaf Width in Pathogen-Inoculated and Non-Inoculated Plots. Different lowercase letters indicate statistically significant differences according to Tukey’s test (*p* < 0.001). C indicates plots without herbicide application (with or without pathogen inoculation).

Statistical evaluation of all sources of variation revealed significant differences among all leaf width parameters and their interactions (Table 4). Compared to the control, an increase in leaf width was observed at the low-dose applications, while higher doses generally also resulted in increased leaf width. In pathogen-inoculated plots, the highest increase (6.1 cm) was recorded with the recommended-dose application of Isoxaflutole + Thiencarbazone-methyl + Cyprosulfamide, representing a 90.63% increase compared to the control. In pathogen-free plots, all three herbicides generally contributed to an increase in leaf width. The noticeable reduction in leaf width in pathogen-inoculated plots compared to pathogen-free plots may be attributed to the effect of the pathogen (Figure 2).

#### 2.5.3. Leaf Length

**Figure 3:**
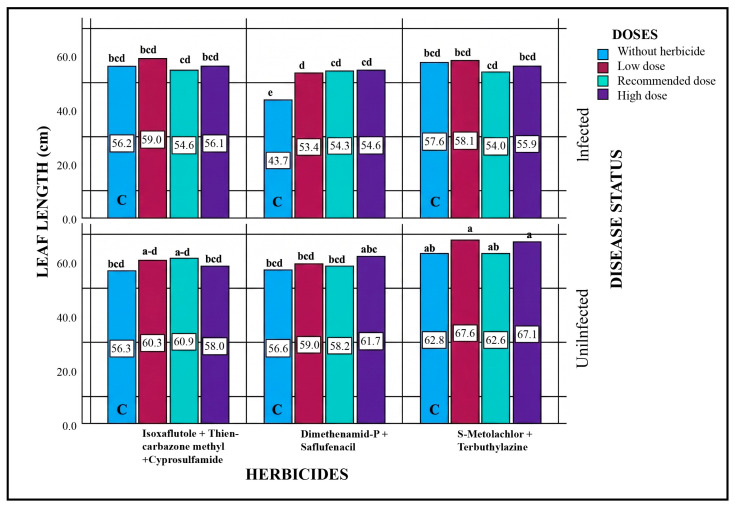
Effects of Different Herbicide Doses on Leaf Length in Pathogen-Inoculated and Non-Inoculated Plots. Different lowercase letters indicate statistically significant differences according to Tukey’s test (*p* < 0.001). C indicates plots without herbicide application (with or without pathogen inoculation).

Statistical evaluation of all sources of variation revealed that, in some cases, significant differences were observed among disease status, herbicide type, dose, and their interactions (disease × herbicide, herbicide × dose, disease × herbicide × dose) (Table 4). In particular, in pathogen-free plots receiving only herbicide applications, all herbicides at all doses generally resulted in increased leaf length compared to the control. Comparing pathogen-inoculated and pathogen-free plots, leaf lengths were shorter in pathogen-inoculated plots, indicating the effect of the pathogen. The lowest leaf length was recorded in the control plot of Dimethenamid-P + Saflufenacil in the pathogen-inoculated area. In pathogen-only plots, leaf length was 43.7 cm, whereas in the low, recommended, and high doses, it increased to 53.4, 54.3, and 54.6 cm, respectively. In the pathogen-free plots for the same herbicide, no significant difference was observed between the low and recommended doses, whereas the high dose resulted in a significant increase. Leaf length in the control was 56.6 cm, rising to 61.7 cm under the high-dose application (Figure 3).

#### 2.5.4. Leaf Number

**Figure 4:**
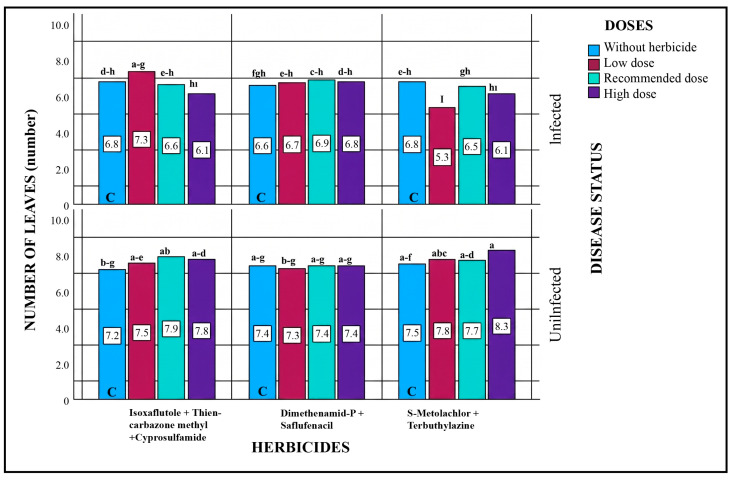
Effects of Different Herbicide Dosages on Leaf Number in Pathogenic and Non-Pathogenic Environments. Different lowercase letters indicate statistically significant differences according to Tukey’s test (*p* < 0.001). C indicates plots without herbicide application (with or without pathogen inoculation).

The effects of different herbicide doses on leaf number were analyzed, and statistical evaluation of all sources of variation revealed that, except for herbicide type and dose, significant differences were observed among other variation sources and their interactions (Table 4). In pathogen-inoculated plots, slight reductions in leaf number were observed in all doses of S-Metolachlor + Terbuthylazine and in the recommended and high-dose applications of Isoxaflutole + Thiencarbazone-methyl + Cyprosulfamide compared to the control. Conversely, the application of Dimethenamid-P + Saflufenacil resulted in a slight increase in leaf number across all doses. In pathogen-free plots, herbicide applications generally caused either an increase or maintenance of leaf number across all doses. Overall, the decrease in leaf number in pathogen-inoculated plots may be attributed to the pathogen, and it is suggested that herbicides, due to their negative effect on the pathogen, do not directly increase leaf number (Figure 4).

#### 2.5.5. Root Collar Diameter

When all sources of variation were evaluated statistically, significant differences in root collar diameter were observed in some treatments for the factors of disease status, herbicide type, dose, and their interactions (disease × herbicide, disease × dose) (Table 5). Comparisons between pathogen-inoculated and pathogen-free conditions indicated an increase in root collar diameter. This increase was more pronounced in pathogen-inoculated plots compared to the control, suggesting that the observed differences in root collar diameter were primarily associated with pathogen presence.

Although root diameters in pathogen + herbicide treatment plots were generally smaller than in non-pathogenic plots, within the pathogen-treated plots, root collar diameters increased relative to the control (pathogen only). This increase is thought to be attributable to the effect of the herbicide. Similar trends were observed across all doses of the three herbicides tested.

**Figure 5:**
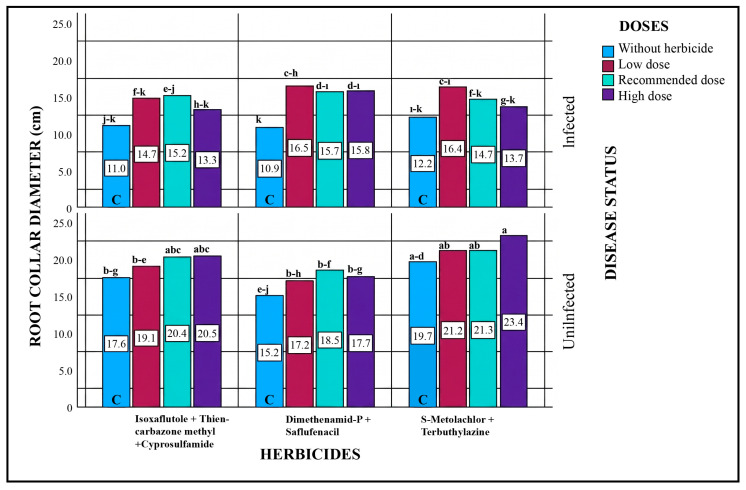
Effects of Different Herbicide Doses on Root Collar Diameter under Pathogenic and Non-Pathogenic Conditions. Different lowercase letters indicate statistically significant differences according to Tukey’s test (*p* < 0.001). C indicates plots without herbicide application (with or without pathogen inoculation).

In non-pathogenic plots treated only with herbicides, root collar diameter increased in all applications. The highest root collar diameter (23.4 cm) was observed at the highest dose of the herbicide containing S-Metolachlor + Terbuthylazine. In comparison, the control plot with the same herbicide had a root collar diameter of 19.7 cm, and the recommended dose yielded 21.3 cm (Figure 5).

#### 2.5.6. Root Length

Statistical evaluation of all sources of variation indicated that, except for the herbicide × dose interaction, significant differences in root length were observed among all other factors and their interactions (Table 5). In pathogen + herbicide treatment plots, herbicide applications generally increased root length compared to the control. This increase was more pronounced at the lower and recommended doses than at the highest dose. However, for S-Metolachlor + Terbuthylazine, root length decreased at the highest dose compared to the control.

**Figure 6:**
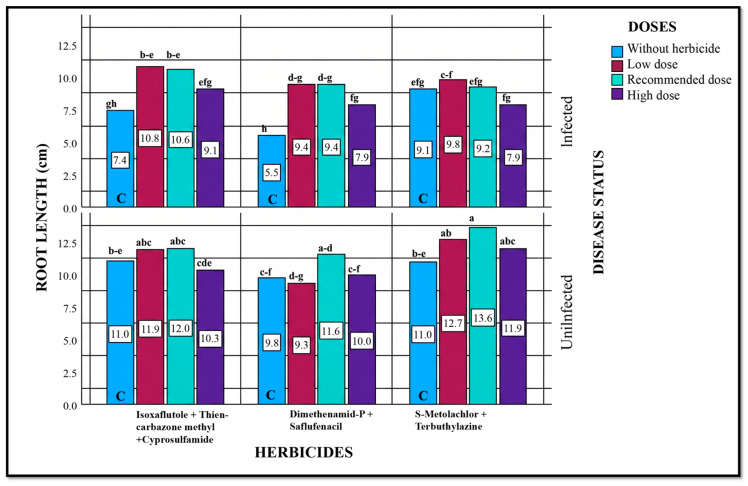
Effects of Different Herbicide Doses on Root Length under Pathogenic and Non-Pathogenic Conditions. Different lowercase letters indicate statistically significant differences according to Tukey’s test (*p* < 0.001). C indicates plots without herbicide application (with or without pathogen inoculation).

In pathogen + herbicide treatments, the maximum root length (10.8 cm) was observed at the lower dose of Isoxaflutole + Thiencarbazone-methyl + Cyprosulfamide, representing a 45.95% increase compared to the control. At the recommended dose of the same herbicide, root length was 10.6 cm, with a 43.24% increase relative to the control. For Dimethenamid-P + Saflufenacil under pathogen treatment, root length also increased significantly compared to the control. In this case, root length in the control was 5.5 cm, while lower and recommended doses produced 9.4 cm, representing a 70.91% increase relative to the control (Figure 6).

Comparisons between pathogenic and non-pathogenic conditions suggest that the observed increases in root length are primarily due to herbicide applications. In non-pathogenic plots (herbicide only), the upper dose of Isoxaflutole + Thiencarbazone-methyl + Cyprosulfamide caused a slight decrease in root length, while other herbicides at all doses did not produce significant differences. The highest root length in non-pathogenic plots was observed with S-Metolachlor + Terbuthylazine at the recommended dose (13.6 cm) (Figure 6).

Overall, the results indicate that herbicide applications were associated with increased root length, likely due to reduced disease pressure.

#### 2.5.7. Root Weight

Statistical evaluation of all sources of variation indicated that significant differences in root weight were observed for disease status, herbicide type, dose, and the disease × herbicide interaction (Table 5). When all treatments were considered, pathogen-only applications caused a significant reduction in root weight compared to the non-pathogenic control. Similarly, in pathogen + herbicide treatments, root weight decreased relative to the control, indicating that this reduction was primarily pathogen-induced.

However, in plots where herbicides were applied together with pathogens, root weight was higher compared to pathogen-only control plots, suggesting a positive effect of herbicide application. In non-pathogenic plots receiving herbicide treatments only, root weight was also higher than in non-pathogenic control plots, further supporting the conclusion that herbicide application increased root weight.

**Figure 7:**
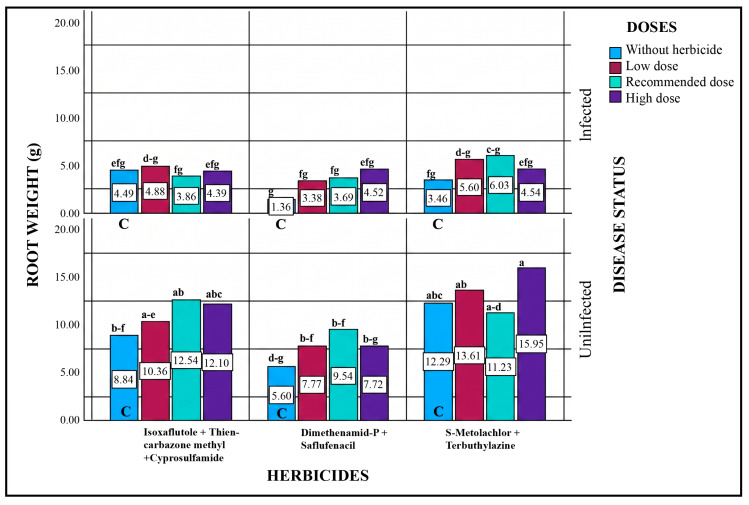
Effects of Different Herbicide Doses on Root Weight under Pathogenic and Non-Pathogenic Conditions. Different lowercase letters indicate statistically significant differences according to Tukey’s test (*p* < 0.001). C indicates plots without herbicide application (with or without pathogen inoculation).

The highest root weight (15.95 g) was recorded in the highest dose of the herbicide containing S-Metolachlor + Terbuthylazine. This represented a 29.77% increase compared to the control (12.29 g). Overall, the results indicate that herbicide applications positively influenced root weight, likely by mitigating the negative effects of pathogens (Figure 7).

## 3. Discussion

In this study, the distribution and pathogenicity of *Fusarium* spp. in maize-production areas of Eskişehir province were investigated, and the effects of commonly used soil-applied herbicides on the development of *F. graminearum* were evaluated under both *in vitro* and *in vivo* conditions. The results revealed that *F. verticillioides* was the most prevalent species, whereas *F. graminearum* exhibited the highest virulence, with a disease severity of 96.67%. These findings are consistent with previous studies reporting the widespread occurrence of *F. verticillioides* in maize and the high pathogenic potential of *F. graminearum* [22,23,24].

Among the identified *Fusarium* species, *F. graminearum* was selected for further herbicide interaction studies because it exhibited both high virulence and prevalence. This selection was based on the aim of evaluating herbicide effects on the most aggressive and epidemiologically relevant pathogen identified in the surveyed maize fields. The inhibitory effects of herbicides observed on *F. graminearum* under *in vitro* conditions should be evaluated in the context of their primary use as weed control agents rather than fungicides. In this context, the effects observed in this study are more likely associated with indirect or non-target influences on fungal growth rather than direct antifungal activity. Previous studies have demonstrated that herbicides can alter soil physicochemical properties and microbial community composition, thereby influencing pathogen development [25,26,27]. In addition, herbicide applications may affect plant physiological responses and stress-related pathways, which can contribute to changes in plant–pathogen interactions [28]. Furthermore, herbicide-induced modifications in rhizosphere interactions have been reported to influence microbial dynamics and plant health [29].

The *in vitro* and *in vivo* experiments demonstrated that the herbicide containing S-Metolachlor + Terbuthylazine showed the highest inhibitory effect against *F. graminearum* compared with the other tested herbicides. *In vitro* assays indicated that the double-dose application resulted in the highest inhibition rate (57.23%), followed by the upper and recommended doses. These results indicate that the tested herbicides can influence fungal growth under certain conditions, although their primary function is weed control. Similar interactions between herbicide application, plant stress responses, and plant–pathogen relationships have also been reported in previous studies investigating plant–microbe interactions in agricultural systems. The dose-dependent increase in inhibition further supports the relationship between herbicide concentration and reduced pathogen impact.

Similarly, other tested herbicides also exhibited increased inhibitory effects with increasing doses, indicating that herbicide dosage plays a critical role in limiting pathogen growth. However, the relatively low inhibition rates observed at lower doses suggest that the inhibitory effect is strongly influenced by both chemical composition and application rate.

Field experiments largely supported the laboratory findings. The herbicide containing S-Metolachlor + Terbuthylazine was again the most effective treatment under field conditions, showing 38.6% and 45.31% inhibition at the recommended and upper doses, respectively. Nevertheless, the inhibitory effects observed in field trials were lower than those obtained under *in vitro* conditions. This difference may be attributed to environmental factors, soil properties, microbial interactions, and the degradation or adsorption of herbicides in the soil environment [30].

The results of this study indicate that certain soil-applied herbicides may contribute not only to weed management but also to the suppression of soil-borne pathogens. However, the potential negative impacts of high-dose applications on crop health, soil microbiota, and the environment should be carefully considered. Therefore, the use of herbicides as supplementary tools for disease management should be evaluated within the framework of integrated pest and disease management strategies.

When examining studies on the identification of *Fusarium* spp. in maize fields in Türkiye, it is observed that the species isolated in this study are similar to those reported in other provinces and regions. A study conducted in Aydın/Türkiye reported that the causal agents of ear rot in maize were *F. moniliforme* and *F. graminearum* [31], while in the Çukurova region, the identified species included *F. graminearum*, *F. moniliforme*, *F. oxysporum*, *F. solani*, and *F. culmorum* [32]. In a similar study conducted in maize fields of the Southern Marmara region, 215 maize samples were collected over different years by the same researchers. The most common and virulent causal agent of stalk and ear rot diseases was identified as *F. verticillioides* (*F. moniliforme*), followed by *F. graminearum* [33]. In this study, 180 samples were collected from Eskişehir, and the most frequently isolated species was *F. verticillioides*, followed by *F. culmorum* and *F. graminearum*. Unlike these previous studies, our study also identified *F. proliferatum*, *F. equiseti*, and *F. acuminatum* from Eskişehir maize fields. Similar results have also been reported in maize fields in different regions of the world. In a study conducted on 9000 asymptomatic maize samples randomly collected from 12 provinces in China, a total of 1022 *Fusarium* isolates belonging to eight different species were identified, including *F. verticillioides* (75.34%), *F. graminearum* (8.32%), *F. proliferatum* (7.14%), *F. subglutinans* (4.11%), *F. meridionale* (1.57%), *F. oxysporum* (1.37%), *F. semitectum* (1.17%), and *F. asiaticum* (0.98%) [22]. Among the species isolated in China, *F. verticillioides*, *F. graminearum*, and *F. proliferatum* were also isolated from Eskişehir. The distribution of *Fusarium* spp. varied across regions, with the highest diversity observed in Hubei province. Similarly, in our study, the isolated species differed among districts: *F. verticillioides*, *F. culmorum*, *F. proliferatum*, *F. graminearum*, and *F. acuminatum* were isolated from all districts, whereas *F. sambucinum* was only found in Alpu, *F. chlamydosporum* only in Odunpazarı, and *F. equiseti* in Odunpazarı and Seyitgazi. In two other studies conducted in China, *F. asiaticum*, *F. equiseti*, *F. graminearum*, *F. meridionale*, *F. proliferatum*, and *F. temperatum*, as well as the *Fusarium incarnatum-equiseti* species complex (14.39%), *F. temperatum* (5.30%), *F. acuminatum* (3.03%), *F. solani* (2.27%), *F. sporotrichioides* (2.27%), *F. tricinctum* (1.52%), *F. asiaticum* (1.52%), *F. verticillioides* (50.00%), *F. subglutinans* (18.94%), and *F. proliferatum* were isolated [23,24] reported *F. graminearum* as the most virulent species in maize, consistent with our findings. Unlike the species isolated in China, our study also identified *F. culmorum* and *F. sambucinum* from Eskişehir. In a study conducted in India in 2023, the most virulent *Fusarium* spp. in maize fields were reported as *F. acutatum*, *F. verticillioides*, and *F. andiyazi*, whereas in our study, the most virulent species was *F. graminearum* [34]. In Italy, in 2023, a study on maize fields reported the isolation of *F. annulatum*, *F. commune*, *F. nisikadoi*, and *F. oxysporum*, all identified as maize pathogens, differing from the species isolated in our study [35]. In a study conducted in South Africa, the *Fusarium* spp. obtained from maize fields through surveys were found to be consistent with the species isolated in our study. The species isolated in that study included *F. verticillioides*, *F. temperatum*, *F. boothii*, *F. subglutinans*, *F. nygamai*, *F. sambucinum*, *F. incarnatum-equiseti*, *F. fujikuroi*, *F. oxysporum*, and *F. chlamydosporum*. Notably, *F. sambucinum* and *F. chlamydosporum*, which were also isolated in our study, were among the species highlighted [36]. These results indicate that *Fusarium* species associated with maize show considerable diversity across different regions, although certain species such as *F. verticillioides* and *F. graminearum* consistently dominate maize-production systems. The frequent occurrence of these species is of particular importance because they are known to cause severe yield losses and produce mycotoxins that threaten grain quality and food safety. Therefore, identifying the dominant *Fusarium* species in a region is essential for developing effective disease management strategies.

In this study, increasing doses of soil-applied herbicides resulted in progressively higher inhibition rates of *F. graminearum*, with the highest efficacy observed in treatments containing S-Metolachlor + Terbuthylazine. Similarly, it was reported that several herbicides were able to suppress *Fusarium* growth under controlled conditions in a dose-dependent manner [37]. These findings suggest that certain herbicide compounds, particularly when applied at higher concentrations, may influence fungal growth under specific conditions. Moreover, previous studies have indicated that herbicide concentration plays a crucial role in shaping interactions with soil microbial communities, especially fungal populations [30]. Soil-applied herbicides may indirectly suppress some soil-borne pathogens by altering microbial community structures, competitive dynamics, and nutrient availability. However, studies evaluating the direct effects of soil-applied herbicides on *Fusarium* species are relatively limited in the literature. Most previous research has focused on systemic herbicides such as glyphosate and their indirect effects on soil microbial communities. Therefore, the findings of the present study contribute additional information on the potential interactions between soil-applied herbicides and *Fusarium* development in maize-production systems. The inclusion of higher and double doses in this study was intended to better characterize the dose–response relationship under controlled conditions. Although higher doses showed greater inhibitory effects under controlled conditions, these results should be interpreted with caution, as such doses may not be agronomically feasible due to potential phytotoxicity and environmental constraints. Therefore, the practical relevance of these findings should be considered primarily within the limits of recommended application rates.

However, the lower efficacy observed under field conditions compared to *in vitro* assays in the present study can be attributed to soil properties, microbial interactions, and chemical degradation processes. It was emphasized that adsorption and transformation processes in soil can limit the direct inhibitory effect activity of herbicides [25]. Similarly, long-term applications of S-Metolachlor have been reported to influence soil microbial diversity, although temporal factors and crop rotations often play a more dominant role than the herbicide itself [38,39]. In addition, prolonged S-Metolachlor application may alter soil microbial biodiversity, which can indirectly affect pathogen suppression through changes in microbial competition and nutrient cycling [40]. These findings support the lower inhibition rates observed in field trials in the present study. Another important outcome of this research is the influence of herbicide application on plant growth parameters. Plants treated with herbicides in the presence of the pathogen generally exhibited better growth than pathogen-only controls, which may be explained by reduced pathogen pressure and modified soil microbial interactions. Herbicides such as terbuthylazine have been reported to affect soil enzymatic activity and microbial communities, thereby influencing nutrient availability and root development [41]. Similar indirect effects have been reported in studies showing that herbicides alter microbial biomass and activity [42]. These interactions may lead to changes in root exudation and rhizosphere processes, which under certain conditions can enhance plant resistance to pathogens.

Furthermore, post-emergence herbicides have been reported to inhibit the *in vitro* growth of *F. oxysporum*, highlighting that fungal sensitivity to herbicides varies according to species, formulation, and environmental conditions [43]. This variability may explain the differences in efficacy observed among the tested herbicides in the present study. While S-Metolachlor + Terbuthylazine showed the strongest inhibitory effect, the other formulations exhibited moderate activity, indicating that chemical composition plays a decisive role in determining inhibitory potential.

Nevertheless, the potential negative effects of herbicides on soil health and non-target organisms should not be overlooked. Alterations in soil microbial communities may reduce populations of beneficial microorganisms that compete with or antagonize pathogens [44]. Excessive or inappropriate herbicide use may disrupt soil biological processes and ecological balance, potentially compromising long-term soil fertility and sustainability [30]. Consequently, although certain herbicides show potential for disease suppression, their use should be carefully optimized within integrated weed and disease management programs.

Overall, the findings of this study highlight the complex interactions among herbicides, soil microorganisms, and host plants. The observed dose-dependent effects and indirect promotion of plant growth under pathogen pressure are consistent with global research indicating that herbicide applications can modify soil microbial communities and influence pathogen suppression. These results emphasize the importance of developing sustainable management strategies that balance effective disease control with the preservation of soil ecosystem health.

However, these findings should be interpreted within the limitations of the present study, and further studies under different environmental conditions are needed to confirm the consistency of these effects. In this context, the suppressive effects of herbicides on *Fusarium* development observed in this study may be associated with several indirect mechanisms. Herbicide applications can alter soil physicochemical properties and microbial community composition, which may influence pathogen survival and activity [25,27]. In addition, the reduction in weed competition following herbicide application may improve plant vigor, thereby enhancing the plant’s ability to tolerate pathogen infection. Some herbicides may also induce physiological responses in plants, including stress-related pathways, which can contribute to increased resistance against pathogens [28]. Furthermore, discrepancies between *in vitro* and field results may be explained by environmental variability, soil interactions, and microbial dynamics that are not fully replicated under controlled conditions. Similar interactions between herbicide application and plant–pathogen relationships have been reported in previous studies [27,28].

## 4. Materials and Methods

### 4.1. Survey and Sampling of Maize Fields

Field surveys were conducted in major maize-production areas of Eskişehir province. Sampling sites were selected to represent different maize-growing locations in the region. Symptomatic maize plants showing typical stalk or ear rot symptoms were randomly collected during the growing season [33].

The surveys were carried out in maize-growing areas of the Seyitgazi, Odunpazarı, Tepebaşı, and Alpu districts using a targeted sampling approach. Sampling intensity was determined according to the maize cultivation area of each district. The geographical coordinates of the surveyed maize fields were recorded using a GPS device. Maize fields were inspected at intervals of approximately 5–10 km. In each field, surveys were conducted along diagonal lines or zigzag transects from the field margins toward the center, and diseased plant samples were collected according to field size [45,46]. Plants showing symptoms such as root, crown, stalk, and ear rot, stunted growth, wilting, or ear deformities at different growth stages were collected and transported to the laboratory for further analyses.

### 4.2. Isolation and Purification of Fungal Isolates

Plant samples transported to the laboratory were stored at 4 °C prior to isolation. Sections were taken from symptomatic root and crown tissues, including both diseased and adjacent healthy areas. The sections were surface sterilized in 1% NaOCl for 1–2 min and rinsed twice in sterile distilled water. After drying between sterile filter papers, the samples were placed on Potato Dextrose Agar (PDA), with 7–8 plant sections distributed across five Petri dishes per sample. The plates were incubated at 25 °C under a 12 h light/12 h dark photoperiod for 7–10 days. Colonies representing different fungi were subcultured onto fresh PDA plates to obtain pure cultures. Single-spore isolations were then performed to obtain axenic cultures. For single-spore isolation, isolates were transferred to Carnation Leaf Agar (CLA) and incubated until sufficient fungal growth was achieved. A conidial suspension was prepared by transferring a small piece of mycelium into sterile distilled water and vortexing for 1–2 min. A drop of the suspension was placed onto Water Agar (WA) and incubated at 25 °C for 18–20 h. After incubation, a single conidium was transferred with a small agar block onto fresh CLA plates and incubated at 25 ± 1 °C to obtain pure cultures [47,48].

### 4.3. Identification of Isolates

Following single-spore isolation, fungi grown on carnation leaf agar were examined microscopically. The isolates were first characterized and identified based on morphological characteristics, including colony morphology and microscopic structures, according to the taxonomic keys described in [47]. Colony morphology and microscopic images of some selected isolates are presented in Supplementary Figure S2. Then, 96 *Fusarium* isolates belonging to the most frequently isolated and highly virunet species (*F. verticillioides*, *F. culmorum*, *F. graminearum*, and *F. proliferatum*) were selected for molecular identification to confirm species identity. Thus, the combination of morphological characterization and molecular confirmation provided reliable identification of the *Fusarium* species. The primers used for molecular diagnostics are presented in Table 6.

Genomic DNA was extracted from approximately 300 mg of mycelium using the CTAB method described by [49]. Mycelial samples were ground in liquid nitrogen and transferred into 2 mL microcentrifuge tubes. CTAB buffer was added, and samples were incubated at 65 °C for 1 h. After chloroform–isoamyl alcohol (24:1) extraction and centrifugation, the aqueous phase was collected, and DNA was precipitated with isopropanol. The DNA pellets were washed with ethanol, air-dried, and resuspended in distilled water. The obtained DNA samples were subsequently used for PCR analyses.

For each PCR reaction with a final volume of 10 µL, the following components were included: 1 µL of genomic DNA (40–50 ng µL^−1^), 5 µL of 2× Master Mix (Vazyme P525), 0.5 µL of forward primer (10 pmol), 0.5 µL of reverse primer (10 pmol), and 3 µL of double-distilled water (ddH_2_O) [50]. PCR products were separated by electrophoresis on 2% agarose gels containing Pronosafe DNA stain (Conda, Spain) and visualized under a UV transilluminator. Amplification with the FUM1-F/FUM1-R, the C51F/C51R and PKS1-F/PKS4-R primers were performed under the following conditions: initial denaturation at 95 °C for 3 min, followed by 35 cycles of 95 °C for 15 s, 59 °C for 15 s, and 72 °C for 15 s, with a final extension step at 72 °C for 5 min [51,52,53]. For the VERT-F1/VERT-F2 primers, PCR amplification was carried out using an initial denaturation at 95 °C for 3 min, followed by 35 cycles of 95 °C for 15 s, 62 °C for 15 s, and 72 °C for 15 s, and a final extension at 72 °C for 5 min [54]. Representative PCR amplification products confirming the molecular identification of the main *Fusarium* species are presented in Supplementary Figure S1. Together with the morphological observations, these results confirm the accuracy of species identification.

**Table 6:** Specific Primers Used for Molecular Identification, Including Their Names, Sequences, and Expected Band Sizes.

Species Name	Primer Name	Primer Sequence	Band Size (bp)	Relevant Reference
*F. verticillioides*	VERT-F1 VERT-F2	GCGGGAATTCAAAAGTGGCC GAGGGCGCGAAACGGATCGG	400	[54]
*F. proliferatum*	FUM1-For FUM1-Rev	ACTTTGCCATTTCCAACCGTAT GGGAGTTTTTCCATCCGAATTT	376	[51]
*F.culmorum*	C51F C51R	ATGGTGAACTCGTCGTGGC CCCTTCTTACGCCAATCTCG	570	[52]
*F.graminearum*	PKS4-F PKS4-R	CGTCTTCGAGAAGAGACAT TGTTCTGCAAGCACTCCGA	280	[53]

### 4.4. Pathogenicity Tests

For the preparation of the inoculum used in pathogenicity tests, wheat bran was placed in 100 mL beakers, moistened with 20–30 mL of water, and sterilized in an autoclave at 121 °C for 1 h on two consecutive days. Fungal isolates grown on PDA were cut into 5 mm diameter disks, and five disks per beaker were transferred into the sterilized wheat bran. The beakers were incubated at 23 ± 2 °C for 15–20 days to allow fungal colonization. After incubation, sterilized pots (10 cm diameter) were filled with a mixture of garden soil, burnt farmyard manure, and fine sand (2:1:1) previously sterilized at 121 °C for 45 min. The inoculum was incorporated into the soil at a rate of 50 g per 1 kg of soil. Three days later, maize seeds of the susceptible cultivar PL538 were surface sterilized in 1% NaOCl for 2 min and sown at a depth of 1 cm, with three seeds per pot and three replicates per treatment. Control pots contained only sterilized soil [55]. Plants were maintained under greenhouse conditions for 25–30 days. Disease severity was then assessed using a modified 0–5 scale [56], where 0 = healthy plants; 1–4 = increasing disease severity (1–10%, 11–30%, 31–50%, and 51–80%, respectively) associated with hypocotyl infection and/or reductions in root and plant length; and 5 = plant death or failure of seed germination. Re-isolation was performed from infected roots. Disease severity was calculated using the Townsend–Heuberger formula [57].

DS = [Σ(*n* × *v*)/(*N* × *V*)] × 100,

where *n* is the number of plants in each disease category, *v* is the disease rating, *N* is the total number of plants assessed, and *V* is the maximum disease rating.

The data obtained from the study, established with three replicates according to a randomized complete block design, were analyzed using the IBM SPSS statistical software package (Version 16). Differences among treatments were determined using analysis of variance (ANOVA) and further evaluated using Tukey’s test.

### 4.5. Effects of Herbicides on Mycelial Growth of the Pathogen Under In Vitro Conditions

In this study, PDA media sterilized by autoclaving at 121 °C for 15 min were supplemented with the active ingredients Isoxaflutole + Thiencarbazone methyl + Cyprosulfamide, Dimethenamid-P + Saflufenacil, and S-Metolachlor + Terbuthylazine at the recommended dose, sub-dose, upper dose, and double dose (Table 7). The experiment was conducted with ten replicates. Sterile distilled water was added to the control plates. From the colonies grown on PDA, 5 mm diameter disks were taken from the actively growing edges using a sterile cork borer. One disk per colony was transferred to the center of each PDA plate containing the herbicide, ensuring direct contact with the medium. The plates were incubated at 25 ± 1 °C. Fungal colony measurements were performed on the 2nd and 3rd days using a digital caliper. Measurements were limited to early growth stages, as colonies in control plates reached full coverage by later stages, making further measurements unreliable. Colony diameter was determined by measuring the vertical and horizontal dimensions of each colony [58]. The effect of herbicides on fungal growth relative to the control (% inhibition) was calculated using the following formula [59]:

% Inhibition, E = (K − M)/K × 100

where: E is inhibition rate (%), K is colony diameter in the control (mm), M is colony diameter in the treatment (mm).

The experiments were conducted to investigate the effects of herbicide active ingredients and their doses on fungal mycelial growth under *in vitro* conditions, and the obtained data were subjected to analysis of variance (ANOVA). Significant differences among treatments were further evaluated using Duncan’s Multiple Range Test and classified according to their levels of significance.

### 4.6. Effects of Herbicides on Disease Severity and Plant Development Under In Vivo Conditions

Field experiments were conducted using the most virulent and prevalent species, *F. graminearum* (M14), identified during pathogenicity tests. The field trials were carried out at the experimental area of the Faculty of Agriculture, Eskişehir Osmangazi University, using an artificial inoculation method. After field inoculation, seeds were sown, and herbicides were applied as pre-emergence treatments according to standard weed control protocols [60] for registered herbicides. After adjusting the spray volumes for each dose, herbicides were applied using a backpack sprayer. The registered soil-applied herbicides used against weeds in the maize plots are presented in Table 8.

Initially, inoculum was prepared for the artificial inoculation experiments. *Fusarium* isolates were grown on PDA medium at 22–24 °C for 7–10 days in an incubator. Subsequently, 3 kg of wheat bran was placed into each of four heat-resistant autoclave bags and moistened with sterile water. The bran cultures were sterilized twice on two consecutive days in an autoclave at 121 °C under 1 atm pressure for 1 h per session. After sterilization, 20 agar discs (1 cm in diameter) obtained from pure *Fusarium* cultures were placed onto each 1 kg portion of bran for inoculation. The bran cultures were incubated for 15 days at 24 °C under a 12 h light/12 h dark photoperiod. To ensure homogeneous fungal growth, the cultures were periodically mixed without opening the bags. For field application, the inoculum was mixed with soil at a ratio of 1:40 and evenly distributed over the experimental plots at a rate of 50 g of inoculated soil per m^2^. After soil inoculation, the plots were regularly irrigated until seed sowing [55].

The experimental field was prepared according to standard cultural practices. Each plot was arranged following standard cereal soil-borne disease trial conditions, with dimensions of 1 × 2 m and 0.5 m safety strips between plots [61]. A total of 72 plots were established, with 25 maize plants per plot. The experiment was conducted in a randomized complete block design with three replicates per treatment. Three different herbicides at four doses were evaluated. Herbicides were applied to the soil one week after pathogen inoculation and one day after sowing. Positive control plots received only the pathogen inoculum, whereas negative control plots were sown with maize seeds without inoculation or herbicide application. Plots were regularly monitored after sowing.

According to standard cereal soil-borne disease trial methodology, treatment effects were evaluated when disease incidence in control plots reached at least 20% [61]. Disease assessments were performed at the 8–10 leaf stage based on root and crown rot symptoms. All disease assessments were performed by a single trained evaluator to ensure consistency. Plants within each plot were classified according to a modified 0–5 disease rating scale based on root infection and reductions in root length and/or plant height [56]. The scale was adapted in this study to include additional symptoms such as root damage and growth reduction, which were dominant and commonly observed. Disease severity (%) was calculated using the Townsend-Heuberger formula. Plant height, leaf number, leaf width, leaf length, collar diameter, root length, and root weight were recorded in all treatment plots. Soil-applied herbicides (Isoxaflutole + Thiencarbazone methyl + Cyprosulfamide, Dimethenamid-P + Saflufenacil, and S-Metolachlor + Terbuthylazine) were applied at the pre-emergence stage according to the manufacturer’s recommended label rates under field conditions. The experiment consisted of herbicide treatments applied to infected and uninfected plots. In the infected plots, the control treatment (C) consisted of plants inoculated with *F. graminearum* but without herbicide application and served as the pathogen control. In the uninfected plots, the control treatment consisted of non-inoculated plants without herbicide application and served as the untreated plant control. At the end of the experiment, fungal re-isolation was performed from plants in all pathogen-inoculated plots.

In *in vivo* studies, disease severity and plant growth parameters (plant height, leaf number, leaf width, leaf length, collar diameter, root length, and root weight) were analyzed using ANOVA based on a randomized complete block design with split plots in IBM SPSS 26 Statistics. Treatment means were compared using Tukey’s multiple comparison test at the 5% significance level.

## 5. Conclusions

This study provides new insights into the distribution and pathogenicity of *Fusarium* spp. in major maize-growing areas of Eskişehir province and evaluates the effects of commonly used soil-applied herbicides on *F. graminearum*. The results showed that *F. verticillioides* was the most prevalent species, while *F. graminearum* was the most virulent pathogen identified in the surveyed maize fields. Among the tested herbicides, the formulation containing S-metolachlor + Terbuthylazine exhibited the strongest inhibitory effect against *F. graminearum* both *in vitro* and under field conditions. In addition, herbicide-treated plots generally showed improved plant growth parameters compared with pathogen-only treatments, suggesting that certain soil-applied herbicides may indirectly suppress disease development. The findings also revealed that herbicide efficacy observed under laboratory conditions was lower under field conditions, emphasizing the influence of environmental and soil-related factors on pathogen–herbicide interactions. Overall, this study highlights the potential role of selected soil herbicides as complementary tools in integrated weed and disease management strategies in maize-production systems. These findings suggest that the interactions between herbicides, soil conditions, and plant–pathogen dynamics should be further investigated to better understand the underlying mechanisms.

## Figures and Tables

**Table 1:** Disease severity values of *Fusarium* spp. obtained from maize fields.

No	Isolate—Species	Disease Severity (%) *
1	M14—*F. graminearum*	96.67 ± 1.53 a
2	M31 D1—*F. culmorum*	93.33 ± 1.52 ab
3	M53 X—*F. culmorum*	93.33 ± 2.89 ab
4	M51 B—*F. graminearum*	90.00 ± 3.00 a–c
5	M8 C1—*F. graminearum*	90.00 ± 1.73 a–c
6	M20—*F. verticillioides*	86.67 ± 1.53 a–d
7	M24—*F. equiseti*	86.67 ± 1.53 a–d
8	M56 Y2—*F. graminearum*	86.67 ± 3.06 a–d
9	M63 C1—*F. verticillioides*	86.67 ± 2.08 a–d
10	M64 C1—*F. verticillioides*	86.67 ± 0.58 a–d
11	M49 X—*F. culmorum*	86.33 ± 2.08 a–d
12	M58—*F. proliferatum*	84.00 ± 2.00 a–d
13	M34 B—*F. graminearum*	83.33 ± 0.76 a–e
14	M61 Y2—*F. culmorum*	83.33 ± 0.58 a–e
15	M64 C—*F. verticillioides*	83.33 ± 0.00 a–e
16	M18—*F. proliferatum*	82.67 ± 2.52 b–e
17	M31 D2—*F. culmorum*	80.00 ± 5.00 b–f
18	M54 Y—*F. proliferatum*	80.00 ± 0.00 b–f
19	M55 W—*F. verticillioides*	80.00 ± 1.00 b–f
20	M56 X2—*F. chlamydosporum*	80.00 ± 1.73 b–f
21	M50 B2—*F. verticillioides*	79.67 ± 2.08 b–f
22	M53 Y—*F. culmorum*	79.67 ± 2.08 b–f
23	M53 E—*F. verticillioides*	76.33 ± 1.15 c–g
24	M33 Y—*F. graminearum*	73.33 ± 3.05 d–h
25	M52 C2—*F. verticillioides*	73.33 ± 5.77 d–h
26	M56—*F. verticillioides*	73.33 ± 5.77 d–h
27	M31—*F. culmorum*	70.00 ± 5.00 e–h
28	M34—*F. culmorum*	70.00 ± 10.00 e–h
29	M50 B1—*F. verticillioides*	70.00 ± 5.00 e–h
30	M53 A2—*F. sambucinum*	70.00 ± 5.00 e–h
31	M55 W2—*F. verticillioides*	70.00 ± 10.00 e–h
32	M44 A—*F. verticillioides*	67.67 ± 6.81 f–h
33	M63 C1—*F. verticillioides*	63.67 ± 6.03 g–i
34	M44 X—*F. verticillioides*	63.33 ± 9.61 g–i
35	M49 B—*F. culmorum*	62.67 ± 2.52 g–i
36	M22 B—*F. acuminatum*	61.67 ± 2.89 h–i
37	M31 Z1—*F. culmorum*	60.00 ± 5.00 h–i
38	M33 X—*F. verticillioides*	60.00 ± 1.00 h–i
39	M33 A—*F. verticillioides*	66.67 ± 5.77 f–h
40	M62—*F. verticillioides*	63.67 ± 6.03 g–i
41	M42 A—*F. acuminatum*	52.67 ± 2.52 i
	Total	76.76 ± 11.29
	F-value	*p* < 0.001

* Values represent the means of three replicates (mean ± SE, *n* = 3). Different letters indicate statistically. Significant differences according to Tukey’s test (*p* < 0.001).

**Table 2:** Inhibition rates of the pathogen by different herbicide doses in Petri dish assays.

Active Ingredient	Dose	Inhibition Rate at First Measurement (%)	Inhibition Rate at Second Measurement (%)	Mean Inhibition Rate (%)
Isoxaflutole + Thiencarbazone methyl + Cyprosulfamide	Sub dose	8.05	6.13	7.09
	Recommended dose	10.9	8.54	9.67
	High dose	13.79	14.45	14.12
	Double dose	17.87	16.67	17.27
Dimethenamid-P + Saflufenacil	Sub dose	20.68	14.51	17.60
	Recommended dose	21.94	17.42	19.68
	High dose	28.58	19.51	20.04
	Double dose	30.87	22.54	26.70
S-Metolachlor + Terbuthylazine	Sub dose	34.05	31.43	32.74
	Recommended dose	38.72	40.18	39.45
	High dose	48.12	46.19	47.15
	Double dose	57.66	56.78	57.22
Control		**-**	**-**	**-**

**Table 3:** Disease severity in maize under field conditions following different herbicide–pathogen treatments.

Herbicide/Dose	Disease Severity (%) *	Efficacy Values (%)
Control	88.27 ± 2.81 f	-
Isoxaflutole + Thiencarbazone methyl + Cyprosulfamide – Sub dose	72.53 ± 4.41 e	17.83
Isoxaflutole + Thiencarbazone methyl + Cyprosulfamide – Recommended dose	68.00 ± 2.88 de	22.96
Isoxaflutole + Thiencarbazone methyl + Cyprosulfamide – High dose	61.60 ± 3.67 bcd	30.21
Dimethenamid-P + Saflufenacil – Sub dose	69.33 ± 3.03 de	21.45
Dimethenamid-P + Saflufenacil – Recommended dose	64.00 ± 2.88 cd	27.49
Dimethenamid-P + Saflufenacil – High dose	57.60 ± 2.17 bc	34.74
S-Metolachlor + Terbuthylazine – Sub dose	65.87 ± 2.31 cde	25.37
S-Metolachlor + Terbuthylazine – Recommended dose	54.67 ± 0.92 ab	38.06
S-Metolachlor + Terbuthylazine – High dose	48.27 ± 3.03 a	45.31
Mean ± SE (SE = Standard Error)	65.01 ± 10.85	
F-value	*p* < 0.001	

* Values represent the mean of three replicates (mean ± standard error, *n* = 3). Different letters indicate statistically significant differences between values according to Tukey’s test at a probability level of *p* < 0.001.

**Table 4:** Analysis of variance showing the effects of different herbicide doses on morphological traits of maize in pathogen-inoculated and non-inoculated plots.

SV	df	Plant Height	Leaf Width	Leaf Length	Leaf Number
Disease Status	1	1802.70 **	47.7346 **	674.149 **	19.9501 **
Herbicide	2	1188.39 **	1.098 *	183.525 **	0.1656 ns
Dose	3	240.88 **	4.2847 **	58.161 **	0.1013 ns
Disease × Herbicide	2	6.00 ns	2.8830 **	64.989 **	1.6335 **
Disease × Dose	3	4.47 ns	1.5749 **	1.246 ns	0.4635 *
Herbicide × Dose	6	45.88 ns	1.6123 **	24.839 *	1.687 *
Disease × Herbicide × Dose	6	22.65 ns	1.5264 **	20.945 *	1.779 **
Error	46	25.53	0.2268	6.123	0.0954

** *p* < 0.01; * *p* < 0.05; ns: not significant; SV: source of variation; df: degrees of freedom.

**Table 5:** Analysis of Variance Table Showing the Effects of Different Herbicide Doses on Root Traits under Pathogenic and Non-Pathogenic Conditions.

SV	df	Root Collar Diameter	Root Length	Root Weight
Disease Status	1	473.585 **	106.5638 **	747.742 **
Herbicide	2	22.422 **	16.1464 **	81.021 **
Dose	3	42.943 **	17.0384 **	16.819 *
Disease × Herbicide	2	35.679 **	3.7713 **	23.411 **
Disease × Dose	3	8.348 **	2.9920 **	2.472 ns
Herbicide × Dose	6	1.687 ns	1.0348 ns	2.210 ns
Disease × Herbicide × Dose	6	1.779 ns	2.7761 **	7.518 ns
Error	46	1.798	0.5236	4.054

* *p* < 0.01, ** *p* < 0.05; ns: Not Significant; SV: Source of Variation; df: Degrees of Freedom.

**Table 7:** *In Vitro* Application Doses of Herbicides Used in the Experiment.

Active Ingredient	Recommended Dose (mL/da)	Sub Dose (µL per Petri dish)	Recommended dose (µL per Petri Dish)	High dose (µL per Petri Dish)	Double dose (µL per Petri Dish)
Isoxaflutole + Thiencarbazone methyl + Cyprosulfamide	35	0.178	0.222	0.267	0.445
Dimethenamid-P + Saflufenacil	100	0.508	0.636	0.763	1.272
S-Metolachlor + Terbuthylazine	500	2.545	3.181	3.817	6.362

**Table 8:** ACTIVE Ingredients and Application Doses Used in *In Vivo* Experiments.

Active İngredient	Recommended Dose (mL/da)	Sub Dose (mL per 2 m^2^)	Recommended Dose (mL per 2 m^2^)	High Dose (mL per 2 m^2^)
Isoxaflutole + Thiencarbazone methyl + Cyprosulfamide	35	0.056	0.07	0.084
Dimethenamid-P + Saflufenacil	100	0.16	0.2	0.24
S-Metolachlor + Terbuthylazine	500	0.8	1.0	1.2

## Data Availability

The original contributions presented in this study are included in the article/Supplementary Materials. Further inquiries can be directed to the corresponding author.

## Supplementary Materials

The following supporting information can be downloaded at: https://www.mdpi.com/article/10.3390/plants15081254/s1, Figure S1: Gel images obtained during the molecular identification of major *Fusarium* species; Figure S2: Morphological characteristics of representative *Fusarium* species used in this study: Colony morphology (A1–D1) and microscopic structures (A2–D2) are shown. (A) *Fusarium graminearum*, (B) *Fusarium culmorum*, (C) *Fusarium verticillioides*, and (D) *Fusarium proliferatum.*
