# Supplementary figures and images for: Characterization of Fusarium Species and Soil Herbicide Effects on Fusarium graminearum in Maize Fields of Eskişehir, Türkiye
Source: Plants (Basel). 2026 Apr 19;15(8):1254. doi: 10.3390/plants15081254 (PMC13120178; doi:10.3390/plants15081254)

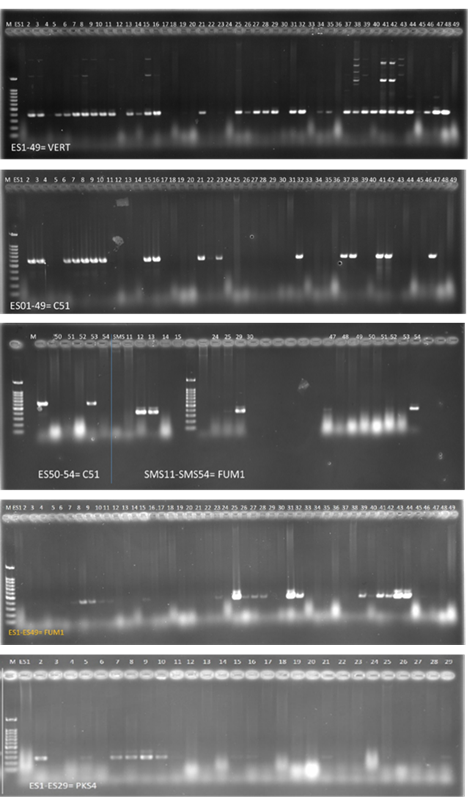

Supplement: Supplementary file 1 [file plants-15-01254-s001.zip › Figure S1.png]

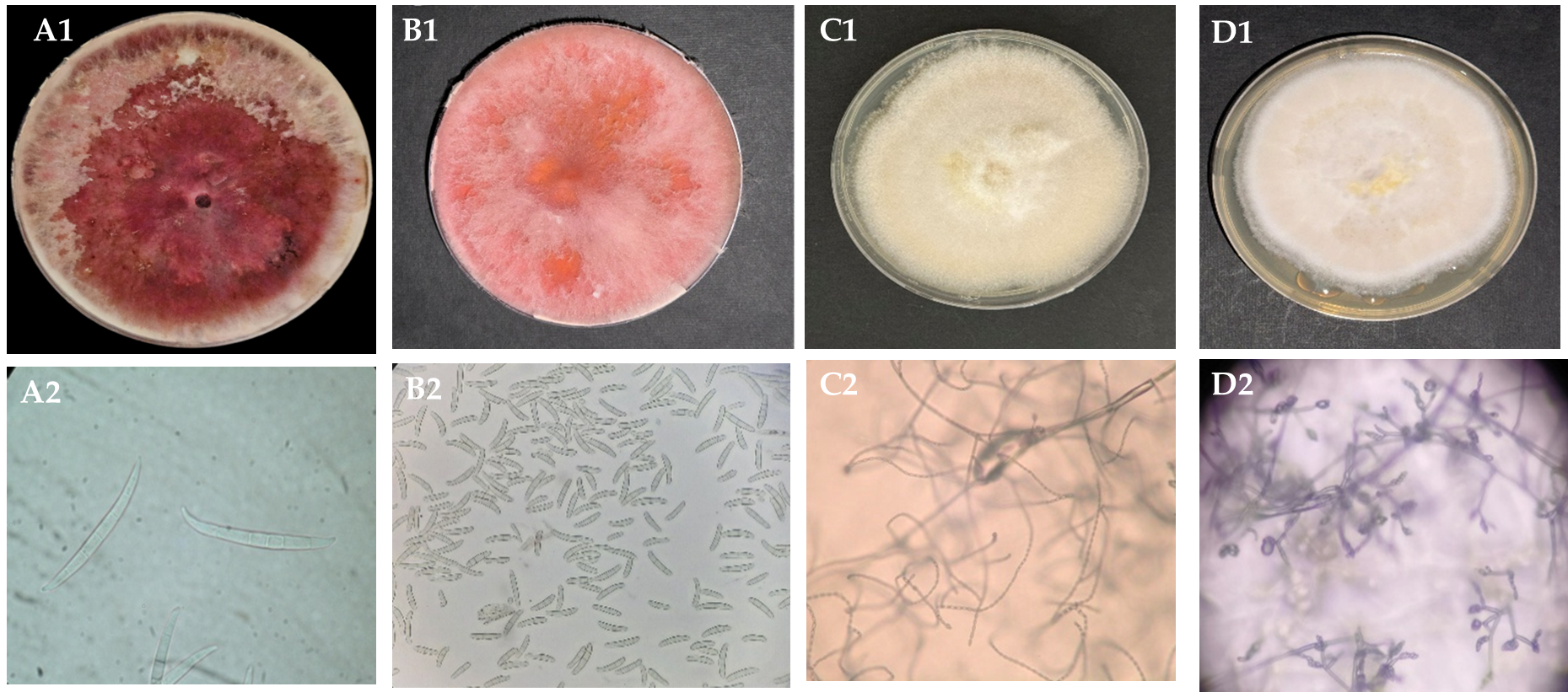

Supplement: Supplementary file 1 [file plants-15-01254-s001.zip › Figure S2.png]
